# Coccidioidomycosis-Related Hospital Visits, Texas, USA, 2016–2021

**DOI:** 10.3201/eid3005.231624

**Published:** 2024-05

**Authors:** Heather Mayfield, Vanora Davila, Elena Penedo

**Affiliations:** Texas Department of State Health Services, Austin, Texas, USA

**Keywords:** coccidioidomycosis, *Coccidioides immitis* infection, *Coccidioides posadasii* infection, Valley fever, fungi, respiratory infections, Texas, United States

## Abstract

We analyzed hospital discharge records of patients with coccidioidomycosis-related codes from the International Classification of Diseases, 10th revision, Clinical Modification, to estimate the prevalence of hospital visits associated with the disease in Texas, USA. Using Texas Health Care Information Collection data for 2016–2021, we investigated the demographic characteristics and geographic distribution of the affected population, assessed prevalence of hospital visits for coccidioidomycosis, and examined how prevalence varied by demographic and geographic factors. In Texas, 709 coccidioidomycosis-related inpatient and outpatient hospital visits occurred in 2021; prevalence was 3.17 cases per 100,000 total hospital visits in 2020. Geographic location, patient sex, and race/ethnicity were associated with increases in coccidioidomycosis-related hospital visits; male, non-Hispanic Black, and Hispanic patients had the highest prevalence of coccidioidomycosis compared with other groups. Increased surveillance and healthcare provider education and outreach are needed to ensure timely and accurate diagnosis and treatment of coccidioidomycosis in Texas and elsewhere.

Coccidioidomycosis, also known as Valley fever, is a fungal disease caused by soil-dwelling *Coccidioides* spp., which include *C. immitis* and *C. posadasii* (*1*,*2*). Coccidioidomycosis is considered a nationally notifiable disease in the United States, but only 26 US states have mandatory reporting requirements at the state level, which has led to incomplete surveillance data across the country (*1*–*3*). Since Centers for Disease Control and Prevention (CDC) reporting began in 1998, the number of Valley fever cases has mostly increased. The steepest increase in the number of cases occurred during 2009–2011, followed by a decrease during 2012–2014 (*4*); prevalence has continued to increase again since 2015 (*4*). Valley fever incidence in Texas, a state where coccidioidomycosis is not reportable, is unknown, but historical and contemporary scientific evidence defines West Texas as a coccidioidomycosis-endemic area (*3*,*5*). In addition, recent climate models suggest that the coccidioidomycosis-endemic region is expanding as temperatures increase and precipitation patterns shift, which might have an indirect effect on the dynamics of this disease in the United States (*6*). Approximately 40% of *Coccidioides* infections are symptomatic, yet not all patients seek medical treatment (*1*,*2*). Symptoms of coccidioidomycosis include cough, fever, and shortness of breath, which might resemble other respiratory illnesses and might be clinically indistinguishable from community-acquired pneumonia (*1*). Coccidioidomycosis can result in life-threatening severe pulmonary or disseminated disease, particularly in groups at high risk, and can also result in chronic illness (*1*–*3*). Surveillance data from Arizona and California, as well as previous research, suggests that demographic factors, such as age, sex, race/ethnicity, and occupation, play a role in a person’s risk for infection and disease complications (*1*–*4*,*7*,*8*). Testing practices vary substantially across states, but overall testing is underused, leading to delayed diagnosis and inadequate treatment (*1*,*2*). 

Although Texas is estimated to be within the geographic range of *C. posadasii*, surveillance data are limited (*3*). We assessed differences in hospital use by patients who had a coccidioidomycosis diagnosis according to demographic and geographic factors. We used data on inpatient hospitalizations and outpatient surgical and radiological procedures from Texas hospitals and ambulatory surgery centers.

## Methods

### Data Source

We obtained study data from inpatient and outpatient public-use data files for January 1, 2016–December 31, 2021, from the Texas Health Care Information Collection (THCIC), Texas Department of State Health Services Center for Health Statistics (*9*,*10*). Institutional review board approval was not necessary to analyze THCIC files. THCIC data include claims for medical services received at hospitals during admission (inpatient) and services received at hospitals without admission (outpatient) from all state-licensed hospitals and ambulatory surgical centers in Texas, except those that are statutorily exempt. Exempt facilities were those located in small counties with populations <35,000 or those located in a county with a population >35,000 but with <100 licensed hospital beds and not in an area designated as urban by the United States Census Bureau. Other exempt facilities were hospitals that did not seek insurance payment or government reimbursement. THCIC public-use data files represent hospital encounters, where 1 encounter contains the final discharge and all related claims information for a deidentified patient, which prevents differentiation between repeat visits. Because public-use data files were used for the analysis, patients could not be identified and deduplicated from the study, which might result in an overrepresentation of persons with severe disease who required multiple visits over time. In this study, hospital visits reflected all inpatient hospitalizations and outpatient surgical and radiologic procedures, as well as ambulatory surgery center visits.

### Study Population

**Figure 1:**
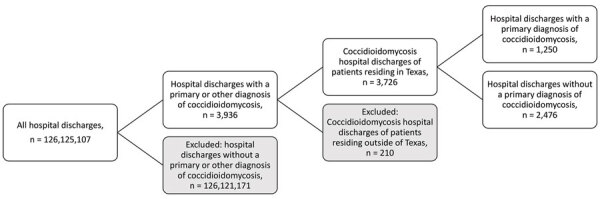
Inclusion and exclusion criteria for study of coccidioidomycosis-related hospital visits, Texas, USA, 2016–2021. Analytic study of patient medical records was conducted to assess prevalence of inpatient and outpatient hospital visits by persons with a coccidioidomycosis diagnosis in Texas. Codes from the International Classification of Diseases, 10th revision, Clinical Modification, were used for diagnoses and included codes B38, B38.0, B38.1, B38.2, B38.3, B38.4, B38.7, B38.8, B38.81, B38.89, and B38.9. Shaded boxes indicate numbers of excluded discharge records and reasons for exclusion from the study. Final analytic study sample was categorized into 2 groups according to clinical diagnostic characteristics.

**Figure 2:**
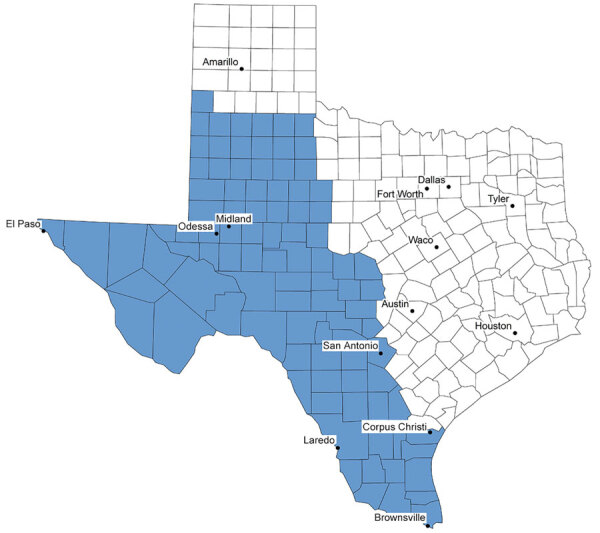
Estimated endemic region of *Coccidioides* spp. fungi in study of coccidioidomycosis-related hospital visits, Texas, USA, 2016–2021. Valley fever region, an estimated 96-county area of Texas determined by using Centers for Disease Control and Prevention Valley fever maps (*5*) spatially overlaid on a Texas county map. Any county that fell within the estimated area was designated as a Valley fever region (blue shading).

To identify the study population, we used codes from the International Classification of Diseases, 10th Revision, Clinical Modification (ICD-10-CM), related to coccidioidomycosis and used all available diagnostic codes within the patient records, including principal and other diagnosis fields (Table 1). We restricted the analytic sample according to the patient’s US state of residence in the record and included only patients with a residence in Texas in our analysis (Figure 1). We identified the exposure status of each patient by using the patient’s county of residence described in the record and the estimated *Coccidioides* spp.–endemic range. We determined the 96 Texas counties estimated to be within the Valley fever–endemic region by using CDC Valley fever maps spatially overlaid on a county map of Texas (Figure 2) (*5*). We designated any county that fell within the CDC’s estimated area as a Valley fever region.

**Table 1:** ICD-10-DM codes used for coccidioidomycosis diagnosis*

Definition	Codes
Coccidioidomycosis	B38
Acute pulmonary coccidioidomycosis	B38.0
Chronic pulmonary coccidioidomycosis	B38.1
Pulmonary coccidioidomycosis, unspecified	B38.2
Cutaneous coccidioidomycosis	B38.3
Coccidioidomycosis meningitis	B38.4
Disseminated coccidioidomycosis	B38.7
Other forms of coccidioidomycosis	B38.8, B38.89
Prostatic coccidioidomycosis	B38.81

*ICD-10-CM, International Classification of Diseases, 10th Revision, Clinical Modification.

### Analysis

We compiled descriptive statistics of all coccidioidomycosis-related hospital visits, including demographic counts and percentages and geographic areas according to discharge year and inpatient/outpatient hospital visits. We also compiled select clinical diagnostic characteristics of the analytic sample as counts and percentages according to hospital visit type (inpatient/outpatient). We calculated prevalence of coccidioidomycosis-related hospital visits as the number of coccidioidomycosis visits per 100,000 hospital visits for any cause and included annual and stratified prevalence according to disease-endemic region status for each year. We conducted a negative binomial regression analysis to calculate prevalence ratios of coccidioidomycosis-related hospital visits for demographic and geographic groups and to calculate 95% CIs. We chose negative binomial regression analysis for all demographic and geographic variables because the data were overdispersed. We added an offset variable calculated as the log of the total hospital visits for any cause to the model to account for the underlying population differences. We included the demographic variables sex and race/ethnicity in the model after determining they were a good fit by using the likelihood ratio test. We included non-Hispanic White, female, and the non–Valley fever–endemic region as reference groups. We conducted all analyses by using SAS version 9.4 software (SAS Institute, https://www.sas.com).

## Results

Of the 3,276 hospital visits representing all inpatient hospitalizations, outpatient surgical and radiologic procedures, and ambulatory surgery center visits for coccidioidomycosis among Texas residents, the percentages of visits were highest among patients who were 46–64 years of age (40.8%), male (56.3%), and Hispanic (47.5%) (Table 2). In 2021, a total of 709 coccidioidomycosis-related hospital visits occurred in Texas (Table 2). Although the 96-county Valley fever region in Texas only accounts for ≈24.0% of the state’s population, those counties had 63.4% of coccidioidomycosis-related hospital visits (Table 2). Inpatient hospital encounters constituted the largest (56.7%) percentage of coccidioidomycosis-related visits (Table 3). The percentage of hospital visits where coccidioidomycosis was the principal diagnosis was only 33.5% (Table 3). When stratified by visit type, only 19.8% of hospital visits that coded as inpatient resulted in a principal coccidioidomycosis diagnosis compared with 51.6% of hospital visits coded as outpatient (Table 3). For inpatient hospital visits where coccidioidomycosis was not the principal diagnosis, many of the diagnosis codes were related to symptoms of severe coccidioidomycosis infection, including sepsis, pneumonia, and respiratory failure. When assessing the principal diagnosis codes for outpatient hospital visits where coccidioidomycosis was not the primary diagnosis, most were related to abnormal diagnostic findings in the lungs or to mild symptoms, such as cough, throat pain, headaches, and abnormal breathing.

**Table 2:** Patient demographics according to year in study of coccidioidomycosis-related hospital visits, Texas, USA, 2016–2021*

Demographics	Year						Total patients
	2016	2017	2018	2019	2020	2021	
No. patients	634	582	571	640	590	709	3,726 (100.0)
Age group, y							
0–17	27	18	26	10	19	27	127 (3.4)
18–44	192	169	157	179	191	189	1,077 (28.9)
46–64	275	244	237	255	233	278	1,522 (40.8)
65–74	86	111	108	127	91	133	656 (17.6)
>75	54	40	43	69	56	82	344 (9.2)
Patient sex							
M	358	363	312	340	335	389	2,097 (56.3)
F	202	150	208	227	202	239	1,228 (33.0)
Unknown	74	69	51	73	53	81	401 (10.7)
Race/ethnicity							
Hispanic	323	301	267	272	252	354	1,769 (47.5)
Non-Hispanic Black	75	65	60	69	79	93	441 (11.8)
Non-Hispanic other†	31	49	51	41	37	41	250 (6.7)
Non-Hispanic White	205	165	193	258	222	221	1,264 (33.9)
Unknown	0	2	0	0	0	0	2 (0.1)
Valley fever region status‡							
Valley fever region	449	388	359	391	360	416	2,363 (63.4)
Non–Valley fever region	185	194	203	238	227	288	1,335 (35.8)
Unknown	0	0	9	11	3	5	28 (0.8)

*Values are no. (%). International Classification of Diseases, 10th Revision, Clinical Modification, codes were used to determine a coccidioidomycosis diagnosis and included codes B38, B38.0, B38.1, B38.2, B38.3, B38.4, B38.7, B38.8, B38.81, B38.89, and B38.9. †Non-Hispanic other category includes American Indian/Eskimo/Aleut, Asian or Pacific Islander, and other. If a hospital had <10 patients of 1 race, that race was categorized as other, and ethnicity of those patients was suppressed. ‡The 96-county area of Texas estimated to be coccidioidomycosis-endemic was determined by using Centers for Disease Control and Prevention Valley fever maps spatially overlaid on a Texas county map. Any county that fell within the estimated area was designated as a Valley fever region.

**Table 3:** Demographics of inpatients and outpatients with coccidioidomycosis in study of coccidioidomycosis-related hospital visits, Texas, USA, 2016–2021*

Demographics	No. inpatients	No. outpatients	Total no. (%)
No. patients	2,114	1,612	3,726 (100.0)
Primary coccidioidomycosis diagnosis			
Yes	418	832	1,250 (33.5)
No	1,696	780	2,476 (66.5)
Age group, y			
0–17	58	69	127 (3.4)
18–44	633	444	1,077 (28.9)
46–64	865	657	1,522 (40.9)
65–74	359	297	656 (17.6)
>75	199	145	344 (9.2)
Patient sex			
M	1,156	941	2,097 (56.3)
F	607	621	1,228 (33.0)
Unknown	351	50	401 (10.8)
Race/ethnicity†			
Hispanic	1,073	696	1,769 (47.5)
Non-Hispanic Black	204	237	441 (11.8)
Non-Hispanic other‡	129	121	250 (6.7)
Non-Hispanic White	708	556	1,264 (33.9)
Geographic region§			
Valley fever region¶	1,395	968	2,363 (63.4)
Non–Valley fever region	700	635	1,335 (35.8)

*International Classification of Diseases, 10th Revision, Clinical Modification, codes were used to determine a coccidioidomycosis diagnosis and included codes B38, B38.0, B38.1, B38.2, B38.3, B38.4, B38.7, B38.8, B38.81, B38.89, B38.9. †Unknown race/ethnicity category was suppressed because the Texas Health Care Information Collection rules dictate any value <10 must be suppressed. ‡Non-Hispanic other category also includes American Indian/Eskimo/Aleut, Asian or Pacific Islander, and other. If a hospital has <10 patients of 1 race, that race is changed to other, and the ethnicity of patients of that race is suppressed. §Unknown geographic region category was suppressed because the Texas Health Care Information Collection rules dictate any value <10 must be suppressed. ¶The 96-county area of Texas estimated to be coccidioidomycosis-endemic was determined by using Centers for Disease Control and Prevention Valley fever maps spatially overlaid on a Texas county map. Any county that fell within the estimated area was designated as a Valley fever region.

**Figure 3:**
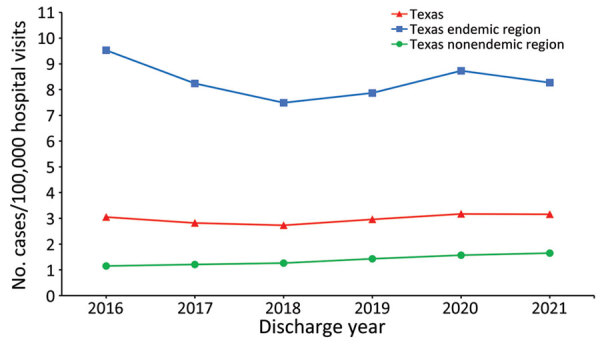
Annual prevalence of inpatient and outpatient hospital visits in study of coccidioidomycosis-related hospital visits, Texas, USA, 2016–2021. Codes from the International Classification of Diseases, 10th Revision, Clinical Modification, were used for diagnoses and included codes B38, B38.0, B38.1, B38.2, B38.3, B38.4, B38.7, B38.8, B38.81, B38.89, and B38.9. Prevalence, defined as the number of Valley fever cases per 100,000 inpatient and outpatient hospital visits for any cause, is indicated statewide by geographic region for each year. Estimated Valley fever–endemic region is a 96-county area of Texas determined by using Centers for Disease Control and Prevention Valley fever maps (*5*) spatially overlaid on a Texas county map. Any county that fell within the estimated area was designated as a Valley fever region.

Prevalence of coccidioidomycosis-related hospital visits was highest in 2020 at 3.17 cases/100,000 hospital visits for any cause and lowest in 2018 at 2.73 cases/100,000 hospital visits for any cause (Figure 3). Prevalence for the estimated Valley fever–endemic region was ≈6–8 times higher than that for the nonendemic region (Figure 3). Although prevalence in the Valley fever–endemic region has decreased since 2016, coccidioidomycosis prevalence in the nonendemic region has steadily increased year to year, starting at 1.15 cases/100,000 hospital visits for any cause in 2016 to 1.65 cases/100,000 hospital visits for any cause in 2021 (Figure 3).

Negative binomial regression analysis suggested that Valley fever region status, patient sex, and race/ethnicity were associated with increased hospital visits for coccidioidomycosis (Table 4). The prevalence ratio (PR) for coccidioidomycosis in men was ≈3 times higher (PR 2.81 [95% CI 2.43–3.25]) than in women. Patients who were non-Hispanic Black (PR 1.51 [95% CI 1.24–1.84]) and Hispanic (PR 1.25 [95% CI 1.05–1.48]) had higher PRs for coccidioidomycosis than those who were non-Hispanic White (Table 4).

**Table 4:** Patient demographics and prevalence ratios for coccidioidomycosis-related hospital visits, Texas, USA, 2016–2021*

Demographics	PR (95% CI)	p value
Patient sex		
F	Referent	NA
M	2.81 (2.43–3.25)	<0.01
Race/ethnicity		
Non-Hispanic White	Referent	NA
Non-Hispanic Black	1.51 (1.24–1.84)	<0.01
Non-Hispanic other†	0.97 (0.78–1.19)	0.74
Hispanic	1.25 (1.05–1.48)	0.01
Geographic region		
Non–Valley fever region	Referent	NA
Valley fever region‡	5.79 (5.03–6.66)	<0.01

*International Classification of Diseases, 10th Revision, Clinical Modification, codes were used to determine a coccidioidomycosis diagnosis and included codes B38, B38.0, B38.1, B38.2, B38.3, B38.4, B38.7, B38.8, B38.81, B38.89, B38.9. p values <0.01 were considered significant. NA, not applicable; PR, prevalence ratio. †Non-Hispanic other category also includes American Indian/Eskimo/Aleut, Asian or Pacific Islander, and other. If a hospital has <10 patients of 1 race, that race is changed to other, and the ethnicity of patients of that race is suppressed. ‡The 96-county area of Texas estimated to be coccidioidomycosis-endemic was determined by using Centers for Disease Control and Prevention Valley fever maps spatially overlaid on a Texas county map. Any county that fell within the estimated area was designated as a Valley fever region.

## Discussion

The lack of surveillance in Texas has led to incomplete knowledge and understanding of the dynamics of coccidioidomycosis. Without surveillance, the burden of disease can only be estimated through indirect metrics. Although the number of coccidioidomycosis-related hospital visits have increased over time, prevalence of visits varied year to year. Those differences might be from fluctuations in the population’s health-seeking behaviors throughout Texas, such as the decline in all-cause inpatient and outpatient hospital visits during the COVID-19 pandemic (*11*) and changes in disease reporting practices and might not be reflective of a change in disease dynamics. However, the observed numbers of hospital visits are likely an underestimate of the true number of coccidioidomycosis cases in Texas because misdiagnosis by physicians, underreporting of cases, and low awareness of coccidioidomycosis might be influenced by geographic location and other patient sociodemographic factors. In addition, only 40% of infections are symptomatic and most symptoms might be indistinguishable from common respiratory illnesses; subsequently, many patients with mild cases or symptoms might not seek medical care (*1*,*2*). The prevalence of coccidioidomycosis in counties outside of the Valley fever region in Texas have been increasing each year, which highlights the need for increased clinician awareness of the disease throughout the state.

Hospital encounters where the principal recorded diagnosis was coccidioidomycosis-related differed substantially between inpatient and outpatient visits, which might have been partly because of different diagnostic practices or medical coding differences for inpatient versus outpatient facilities (*12*). However, patients seeking inpatient care tended to have principal diagnosis codes for conditions related to severe coccidioidomycosis, whereas patients seeking outpatient care had principal diagnostic codes related to mild coccidioidal syndromic symptoms. Therefore, principal diagnostic codes for inpatient and outpatient settings might have reflected differences in disease severity between the 2 patient populations (*12*,*13*).

National surveillance data for coccidioidomycosis include only the 26 mandatory reporting states, and most cases are from Arizona and California, where coccidioidomycosis has increased since 2000 (*4*). National data from CDC only include data during 1998–2019 and show that case counts have fluctuated through the years (*4*). Additional data from California indicate that case counts decreased from 9,000 in 2019 to 7,000–8,000 during 2020–2022 (*14*). In Arizona, new cases increased from 10,358 cases in 2019 to >11,400 cases each in both 2020 and 2021 but then declined to 9,515 cases in 2022 (*15*). Compared with national trends in new cases, Texas displays similar oscillating patterns in hospital visits related to coccidioidomycosis. We found a significant association between coccidioidomycosis-related hospital visits and patient sex or race/ethnicity (p<0.01). Consistent with national surveillance data and previous research, most coccidioidomycosis-related hospital visits in Texas were by male patients who had a higher coccidioidomycosis prevalence than female patients. In Texas, coccidioidomycosis-related hospital visit counts were highest among Hispanic persons, which might reflect the proportion of Hispanic persons residing in the Valley fever–endemic region. According to data from the Texas Demographic Center’s population projections, the percentage of Hispanic persons within the Valley fever–endemic region is ≈68% versus 30% within the nonendemic region (*16*). The increased percentage of Hispanic persons in the Valley fever–endemic region likely contributes to the higher coccidioidomycosis-related hospital visit counts in this group overall. Those findings differ from national surveillance data and previous research that show the number of cases is highest in non-Hispanic White persons but varies between states. Prevalence of coccidioidomycosis-related hospital visits was highest in non-Hispanic Black persons compared with other race/ethnicity groups, which aligns with previous research that suggests non-Hispanic Black persons are at increased risk for infection and severe disease.

Although only 96 counties make up the estimated Valley fever–endemic region within Texas, this area might continue to expand because of shifts in climate-related factors, such as increased temperatures and shifts in precipitation patterns throughout the state that create a more suitable climate for *Coccidioides*. As the Valley fever–endemic region expands in Texas, increased surveillance by public health authorities and increased disease awareness by physicians and healthcare professionals will be critical for monitoring disease spread to susceptible populations and for addressing the health needs of the Texas population.

Although coccidioidomycosis is not a reportable disease in Texas, a substantial disease burden is likely affecting the population, as seen by the number of hospital visits for the disease across the state. In addition, we found that certain groups have higher prevalence, which creates health differences within the Texas population and might lead to worse health outcomes for those groups. Coccidioidomycosis is a serious public health concern; the disease can be difficult to prevent, diagnose, and treat. Disease awareness among local public health officials, physicians, healthcare professionals, and the public is critical to ensure persons seek care when infected and healthcare providers can correctly identify and manage the condition. We assessed exposure according to the patient’s county of residence, but this method might not be a true reflection of where patients were exposed or where they might seek medical care. Therefore, healthcare providers across Texas need to be aware of coccidioidomycosis and the geographic range of *Coccidioides* fungi and inquire about work and travel to determine potential exposures to *Coccidioides* during patient intake.

Our findings indicate that increased testing for coccidioidomycosis should be performed in Texas for patients with pneumonia of unknown cause to prevent delayed diagnosis and ensure prompt disease management and treatment, especially for patients who live in or have traveled to the 96-county disease-endemic area. Because coccidioidomycosis infections can manifest as common respiratory illnesses, clinicians should consider coccidioidomycosis testing in addition to bacteria and virus testing as part of regular diagnostic practices for patients manifesting symptoms consistent with coccidioidomycosis. CDC has developed a clinical testing algorithm for coccidioidomycosis to aid clinicians in diagnosing patients who manifest nonspecific respiratory symptoms similar to community-acquired pneumonia (*17*).

The first limitation of our study is that THCIC suppression rules dictate that gender is suppressed for any patient with a diagnosis code determined to be of a sensitive nature (e.g., drug or alcohol-related or HIV diagnosis). The suppression rule affected ≈401 (≈10%) records in the analytic study sample, which might have affected the final analysis results, including prevalence ratios. Second, race and ethnicity are required to be reported to THCIC by law, but those variables are not generally collected by hospitals and might be subjectively captured in the record by medical staff. Because of the subjective nature of the race and ethnicity data, patients might have been miscategorized by hospital staff, potentially affecting final analyses. Third, county of residence is not collected by hospitals; instead, Federal Information Processing Standards codes are assigned to counties by the Texas Department of State Health Services according to patients’ postal (ZIP) codes, which might be inaccurate for codes that cross county lines and lead to an inaccurate exposure classification in the patient record. Fourth, because the study used public-use data files for inpatient and outpatient records, patients could not be identified and deduplicated from the study sample, which might have resulted in outcome overestimation. Fifth, the nature of medical discharge data might also lead to substantial miscoding or undercoding by medical billing staff, resulting in a potential underestimation of coccidioidomycosis in this study. Finally, the lack of coccidioidomycosis surveillance in Texas might have led to underreporting and underdiagnosis by physicians because of decreased awareness, furthering potential underestimation of case numbers in this study.

In conclusion, coccidioidomycosis-related hospital visits were highest in the estimated Valley fever region of Texas, but ≈33% of visits were in counties located in the non–Valley fever–endemic region, highlighting the need for increased awareness of the disease across the state. Monitoring trends in inpatient and outpatient hospital visits for coccidioidomycosis in medical discharge data and other available data streams will be critical to identify potential new areas of disease endemicity. Monitoring additional data sources for coccidioidomycosis cases in Texas can help close the surveillance gap in the state and increase understanding of disease dynamics. As the Valley fever–endemic region expands, increased surveillance and healthcare provider education and outreach concerning the disease will be needed to ensure timely and accurate diagnosis and treatment.
